# Supplementary material for: Long-range depth imaging using a single-photon detector array and non-local data fusion
Source: Sci Rep. 2019 May 30;9:8075. doi: 10.1038/s41598-019-44316-x (PMC6542841; doi:10.1038/s41598-019-44316-x)
Supplement: Supplementary file 1 — Supplementary Info [file 41598_2019_44316_MOESM1_ESM.pdf]

Supplementary Information: Long-range depth imaging using a single-photon detector array and non-local data fusion

Susan Chan[1], Abderrahim Halimi[1], Feng Zhu[1], Istvan Gyongy[2], Robert K. Henderson[2], Richard Bowman[3], Steve McLaughlin[1], Gerald S. Buller[1] and Jonathan Leach[1]

[1] *School of Engineering and Physical Sciences, Heriot-Watt University, Edinburgh, EH14 4AS, UK*

[2] *Institute for Integrated Micro and Nano Systems, The University of Edinburgh, Edinburgh, EH9 3JL, UK and*

[3] *Department of Physics, University of Bath, Bath, BA2 7AY, UK*

I. SUPPLEMENTARY INFORMATION

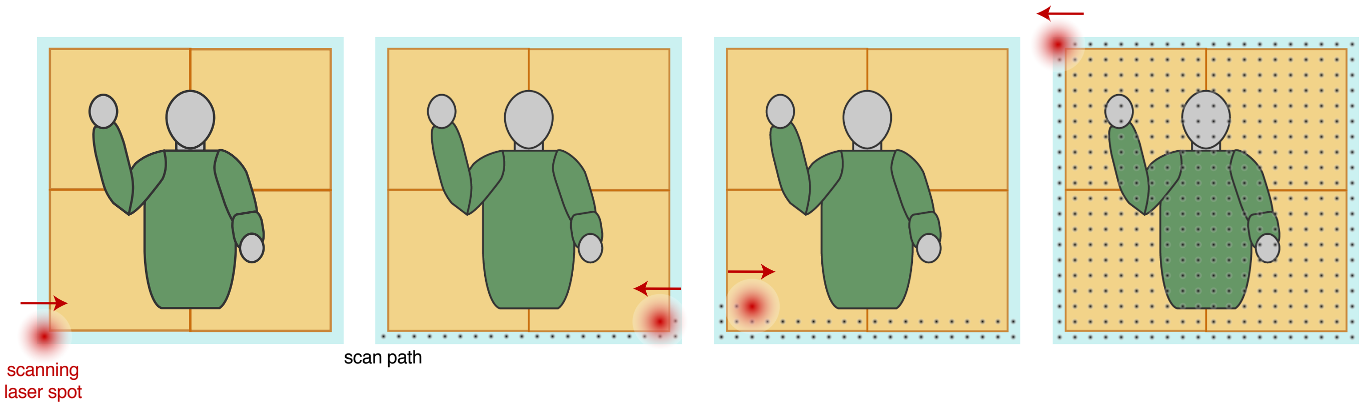

FIG. 1. Illustration of the scan path taken by the laser illumination during the data acquisition. The laser is scanned along a path of 20 by 20 positions, and the illumination is set to cover around a 50 by 50 pixel area. The data from the 20 by 20 scan is combined to produce a single image, corresponding to a particular gate setting. The gate is then scanned in order to produce data for a three-dimensional image.

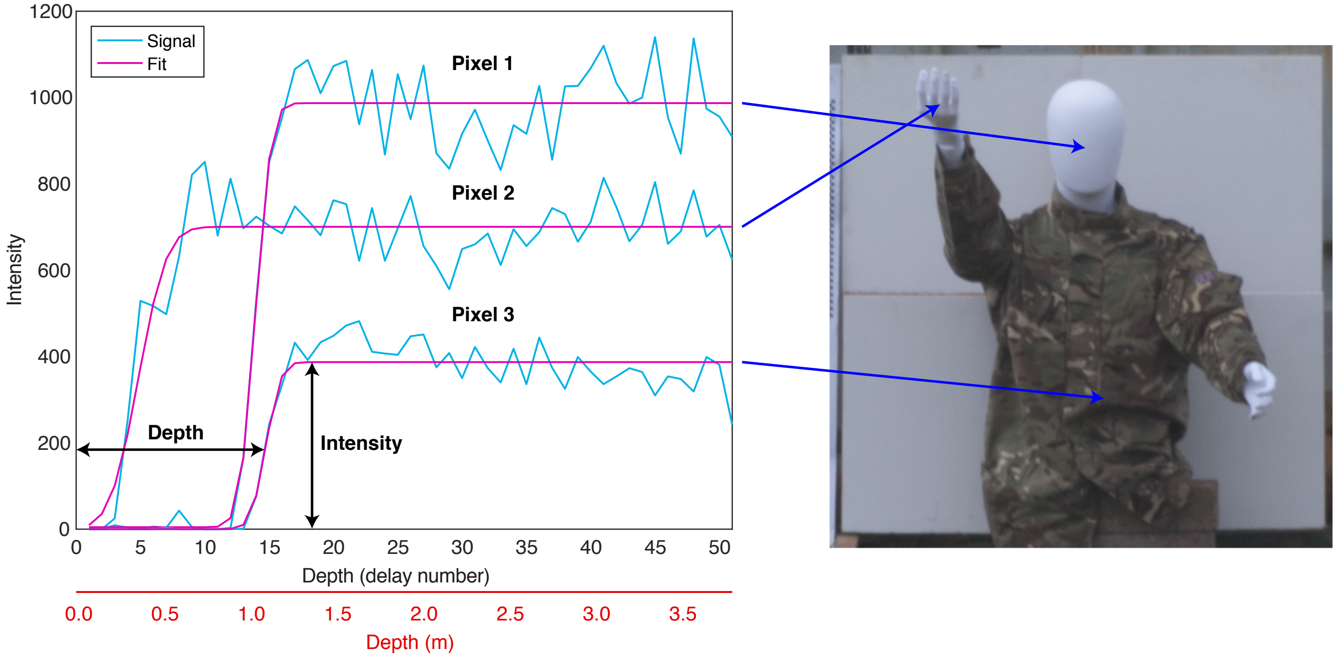

FIG. 2. Sample fit of the error function to the intensity data recorded by three pixels on the SPAD sensor array. Correction for temporal mismatch has not been applied. The blue line is the data after preprocessing and the magenta line is the fit. The position of the targets can be established to a precision within 1 cm.

A. Data preparation

The two-dimensional image for a particular depth is constructed from individual image frames captured by performing a 20 by 20 scan of the target scene, see Fig. 1. The first 30 frames of each dataset of 400 frames are discarded as the frames are captured before the SPAD sensor array is fully initialized. A further 20 frames at the end of each dataset are discarded since the frames do not contribute to the three-dimensional (3D) reconstruction of the scene. By removing these redundant frames, we reduce the computation time. The remaining 350 frames for each fixed

depth are then divided into 175 pairs made up of each laser scan position in the upper half of the array and the corresponding scan position in the lower half. For example, $(1,176),(2,177),\cdots,(175,350)$.

The initial step of our preprocessing is to subtract the noise that originates from the isolated hot pixels with very high dark count rates. For each frame in the pair, we apply hot pixel compensation (*inpaint_nans* function for Matlab) wherein we replace hot pixel elements in the array with an 8-neighbor average. Here, we define a hot pixel as one with a dark count higher than 20% of the total number of bit planes added.

Next, we filter out the noise that comes from daylight illumination of the target scene. We obtain the absolute value of the difference between the two hot pixel-compensated frames. The result is that the almost invariable noise from the sunlight is filtered out while the target signal is preserved. In Eq.(1), fa_i indicates the result after step 2.

$$fa_i = |\text{frame}_i - \text{frame}_{i+175}| \tag{1}$$

We further suppress the noise due to dark counts by applying the Matlab median filter across pixels in a 3-by-3 square neighborhood. This results in pixels that are brighter or darker than those in the neighborhood being replaced by the median value. In Eq.(2), fb_i indicates the result after step 3.

$$fb_i = \text{medfilt2}(fa_i, [3\ 3]) \tag{2}$$

We also want to ignore all pixels for which the intensity is less than a threshold of 10. The laser illuminates a small area in each frame, but the intensity of pixels in the rest of the frame is not exactly zero. To force the intensity of pixels that lie outside the illumination area to zero, we set 10 as a threshold and replace all intensities less than this value with zero for all pixels. For all other pixels, the intensity becomes the original value minus the threshold. In Eq.(3), fc_i indicates the result after step 4.

$$fc_i = |fb_i - \text{threshold}| + (fb_i - \text{threshold}) \tag{3}$$

We carry out these steps for all 175 pairs, after which the noise is suitably filtered out. We then sum all the 175 frames together to obtain a 2D image for the target scene at a given depth sample. In Eq.(4), fd_i indicates the result after this last step.

$$fd = \sum_{i=1}^{175} fc_i \tag{4}$$

The process is repeated for the 240 by 320 by 400 by 51 four-dimensional data cube to obtain the 2D images for the full range of depths measured. Bright pixels in an image indicate that the illuminated objects are inside the corresponding time gate while dark pixels indicate that they are outside.

B. Non-local data fusion

1. *Observation model.*

For each depth location, the proposed LIDAR system scans the scene using N_b beams leading to a photon count image for each depth location. Denoting by $\boldsymbol{y}_{n,k}$ the number of photon counts within the kth depth sample of the nth pixel, and given the acquisition process, the observed data $\boldsymbol{y}_{n,k}$ can be assumed to be distributed according to a Poisson distribution $\mathcal{P}(.)$ as follows

$$y_{n,k} \sim \mathcal{P}(s_{n,k}) \tag{5}$$

where $s_{n,k}$ denotes the average photon counts whose shape is related to the system impulse response (IR) or gate. In this paper, we approximate it using the following parametric formulation

$$s_{n,k} = \frac{r_n}{2}\left\{1 + \text{erf}\left[\frac{k-d_n}{h}\right]\right\} + b_n \tag{6}$$

where erf(.) denotes the error function, $h \geq 0$ is a fixed IR intrinsic parameter which represents the width of the leading edge, and $d_n \geq 0$ and $r_n \geq 0$ are related to the target's depth and intensity, respectively. Here, we assume the absence of background $b_n = 0, \forall\, n$, since the noise has been removed at the preprocessing stage. Figure 2 shows a sample fit to the experimental data. The proposed method aims at estimating the target parameters $\boldsymbol{\Theta} = (\boldsymbol{d}, \boldsymbol{r})$ while considering the observed histograms $\boldsymbol{y}_{n,t}, \forall\, n, t$, and their statistics in Eq.(5). A common approach to achieve this

task is to maximize the likelihood $P(\boldsymbol{Y}|\boldsymbol{\Theta})$ with respect to the parameters, or equivalently to minimize the negative log-likelihood obtained by assuming independence between the observed pixels conditional on $\boldsymbol{\Theta}$, as follows

$$\mathcal{L}(\boldsymbol{\Theta}) = -\log\left[P(\boldsymbol{Y}|\boldsymbol{\Theta})\right] = \sum_n \mathcal{L}_n + constant \tag{7}$$

where

$$\mathcal{L}_n = \sum_{k=1}^{K} \left[s_{n,k} - y_{n,k}\log\left(s_{n,k}\right)\right] \tag{8}$$

where $\boldsymbol{Y}$ is a $K \times N$ matrix gathering the vectors $\boldsymbol{y}_n = (y_{n,1}, \cdots, y_{n,K})$ in its columns, and K is the number of depth samples and N the number of pixels.

2. Fusion-based regularization

Estimating the parameters $\boldsymbol{\Theta}$ in under-sampling conditions is an ill-posed inverse problem that requires the introduction of prior knowledge (or regularization terms) related to the target depths and intensities. In this paper, we propose to solve the following optimization problem

$$\mathcal{C}(\boldsymbol{\Theta}) = \mathcal{L}(\boldsymbol{Md}, \boldsymbol{Mr}) + i_{\mathbb{R}_+}(\boldsymbol{\Theta}) + \tau_1\phi_1^{\boldsymbol{v}}(\boldsymbol{d}) + \tau_2\phi_2^{\boldsymbol{w}}(\boldsymbol{r}) \tag{9}$$

where $\tau_1 > 0$, $\tau_2 > 0$ are two regularization parameters, $\boldsymbol{M}$ is a $Q \times N$ binary matrix that contains a single non-zero value in each row to model the loss of some image pixels due to compressive sampling and Q is the number of non-empty pixels, $i_{\mathbb{R}_+}(\boldsymbol{\Theta})$ is the indicator function imposing non-negativity on the elements of $\boldsymbol{\Theta}$ ($i_{\mathbb{R}_+}(x) = 0$ if x belongs to the non-negative orthant and $+\infty$ otherwise), and $\phi_1^{\boldsymbol{v}}$, $\phi_2^{\boldsymbol{w}}$ are two regularization functions that depend on two weighting vectors $\boldsymbol{v}$ and $\boldsymbol{w}$, respectively. These weights can be evaluated by considering single-photon data, or more interestingly, by adopting a fusion approach. The latter can be performed by learning the weights on other imaging modalities of the same scene, which contain complementary information. The regularization terms and the weights are defined in the following sections.

Prior on parameters. Most image restoration algorithms exploit spatial correlation of the image to reduce the noise and reconstruct missing elements. This can be done by imposing sparsity constraints on image features such as their gradient or their projection using a dictionary. In this paper, we consider a non-local regularization approach as such approaches have shown clear advantages in reconstructing natural images (especially textured images) [1–3]. The proposed regularization term can be expressed as

$$\phi^v(\boldsymbol{x}) = ||\boldsymbol{H}_v\boldsymbol{x}||_F^2 = \sum_{m=1}^{n_d}\sum_{n=1}^{N} v_{m,n}^2 \left(H_m^{\text{Diff}}\boldsymbol{x}\right)^2\Big|_n \tag{10}$$

where $\boldsymbol{H}_v \in \mathbb{R}^{n_d N \times N}$ is a block-circulant-circulant-block matrix (BCCB) which computes weighted differences between each pixel and other n_d pixels located in a predefined field, $\boldsymbol{v} \in \mathbb{R}^{n_d \times N}$ is a matrix of weights associated with each pixel and each direction, and $H_m^{\text{Diff}} \in \mathbb{R}^{N \times N}$ computes the difference between each pixel and that located in the mth direction. This regularization enforces small weighted quadratic differences between similar pixels in the image, i.e. promotes spatial correlation between pixels. The same regularization term Eq.(10) is applied for depth and intensity while considering different weight vectors, as described in the following section.

Weight selection. The weights control the level of regularization imposed on each pair of pixels and should be chosen carefully to improve performance. As previously mentioned, these coefficients can be estimated from the data themselves, or preferably by considering a complementary modality of acquisition. Therefore, in addition to three-dimensional (3D) LIDAR data, we also acquire a co-registered passive RGB optical image of the same scene denoted by $\boldsymbol{z}$ (of size $3 \times N$). The intensity weights $\boldsymbol{w}$ will then be learned on this optical image as follows

$$w_{nm} = \max\left[0.5, \frac{1}{3}\sum_{i=1}^{3}\exp\left(-\frac{|z_{i,n} - z_{i,m}|}{\sigma_w}\right)\right], \tag{11}$$

where we consider an ℓ_1 norm to compute pixel differences to preserve sharpe edges and truncate values smaller than 0.5 to avoid strong downweighting of pronounced edges, as in [4], (σ_w is chosen to be 0.1).

The coefficient $\boldsymbol{v}$ should reflect our knowledge about the target's depth. In this paper, we assume that close regions should share similar depth as for local correlation approaches [5, 6]. Under these considerations, the weights can be evaluated as follows

$$v_{nm} = \frac{1}{x_v \, ||P_n - P_m||_2} w_{nm} \tag{12}$$

where $P_n = (r_n, c_n)$ gathers the row and column coordinates of the nth pixel and x_v is a normalization constant given by the maximum value of $\frac{1}{||P_n - P_m||_2}, \forall \; n, m$. All weighting functions and coefficients in this paper are chosen empirically.

3. Results

This section evaluates the performance of the proposed non-local fusion-based algorithm when considering a compressive sensing scenario, i.e. reducing the number of scan points. The evaluation is carried out on the data presented in the main text. The proposed algorithm is compared to the following:

- Classical algorithm: the depth and intensity parameters are estimated by fitting the IR in Eq.(6) to the data;
- TV algorithm: a total variation prior is applied to Eq.(9) to restore the depth and intensity parameters.

Figure 3 shows that the classical algorithm provides a corrupted depth map when considering 25% of the scanned positions, while the TV algorithm and the one proposed both provide similarly good results owing to their ability to account for spatial correlations (see Fig. 4 and 5 respectively). Further reducing the number of scanned positions leads to a challenging reconstruction problem and TV results are affected when only 10% of the scanned positions are considered. In contrast, the proposed algorithm shows robust results since it uses non-local information and benefits from the complementary information provided by the passive optical camera. This is further highlighted when considering 5% of the scanned positions; the proposed algorithm is able to reconstruct part of the mannequin's left arm, which is missing from the heavily corrupted depth images provided by the other algorithms. 25%, 10% and 5% of scan positions correspond to 83.7%, 61.8%, and 35.8% of pixels respectively. Correction for temporal mismatch has been applied. This shows the clear advantage of the proposed algorithm in data reconstruction under an extremely reduced number of scan positions, i.e. a reduced acquisition time.

---

[1] Buades, A., Coll, B. & Morel, J.-M. A review of image denoising algorithms, with a new one. *SIAM Journal on Multiscale Modeling and Simulation: A SIAM Interdisciplinary Journal* **4**, 490–530 (2005).

[2] Dabov, K., Foi, A., Katkovnik, V. & Egiazarian, K. Image denoising by sparse 3D transform-domain collaborative filtering. In *IEEE Transactions on Image Processing*, 2080–2095 (2007).

[3] Salmon, J., Harmany, Z., Deledalle, C.-A. & Willett, R. Poisson Noise Reduction with Non-local PCA. *Journal of Mathematical Imaging and Vision* **48**, 279–294 (2014).

[4] Lanaras, C., Bioucas-Dias, J., Baltsavias, E. & Schindler, K. Super-Resolution of Multispectral Multiresolution Images from a Single Sensor. In *IEEE Conference on Computer Vision and Pattern Recognition Workshops* (2017).

[5] Rudin, L. I., Osher, S. & Fatemi, E. Nonlinear total variation based noise removal algorithms. *Physica D* **60**, 259–268 (1992).

[6] Iordache, M.-D., Bioucas-Dias, J. M. & Plaza, A. Total Variation Spatial Regularization for Sparse Hyperspectral Unmixing. In *IEEE Transactions on Geoscience and Remote Sensing*, 4484–4502 (2012).

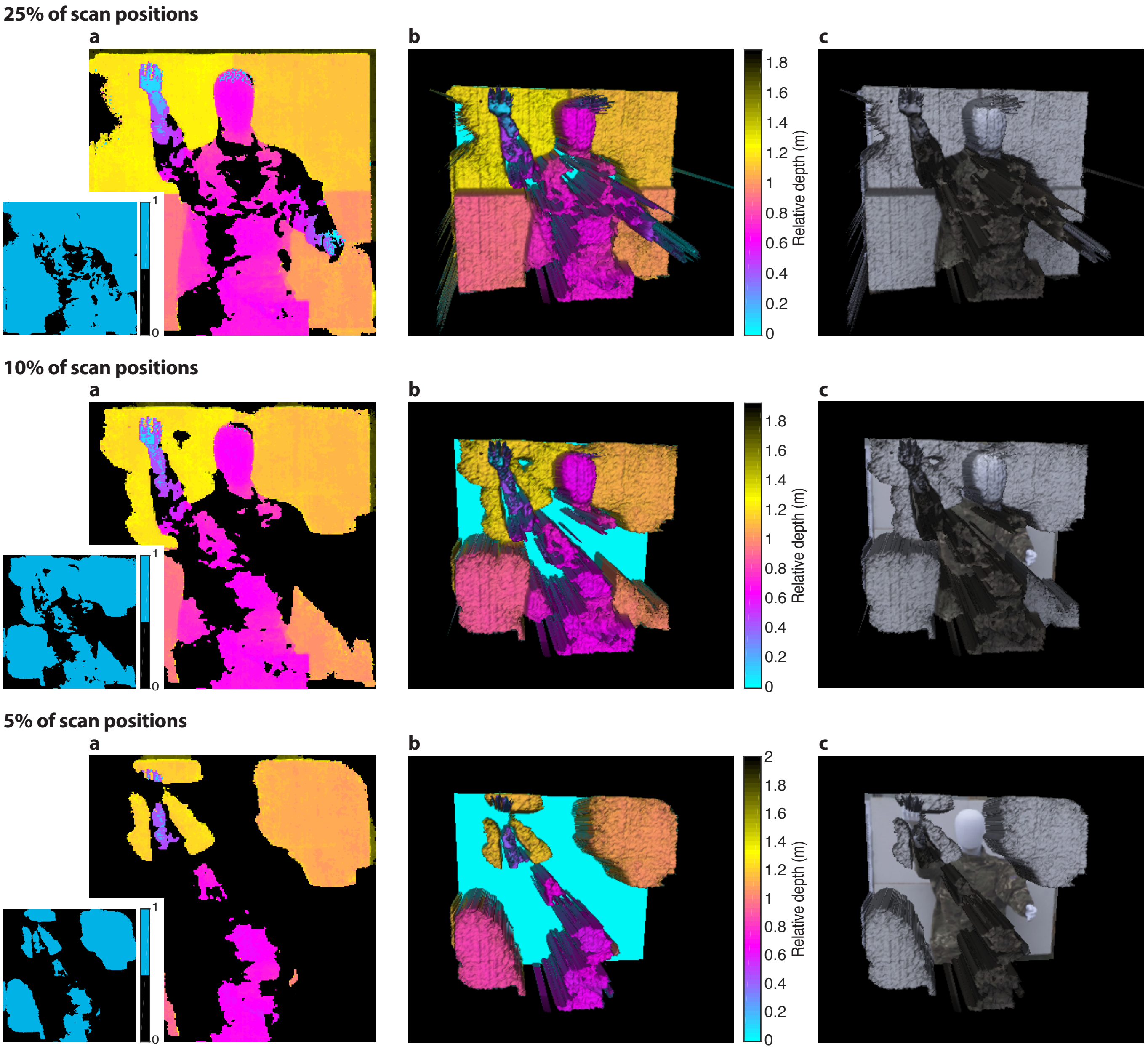

FIG. 3. Reconstructions of the target scene obtained by fitting using 25%, 10%, and 5% of the scanned positions. Retrieved depth information in (a) 2D and (b) 3D. (c) The intensity information from a DSLR camera overlaid on top of the retrieved depth information in 3D.

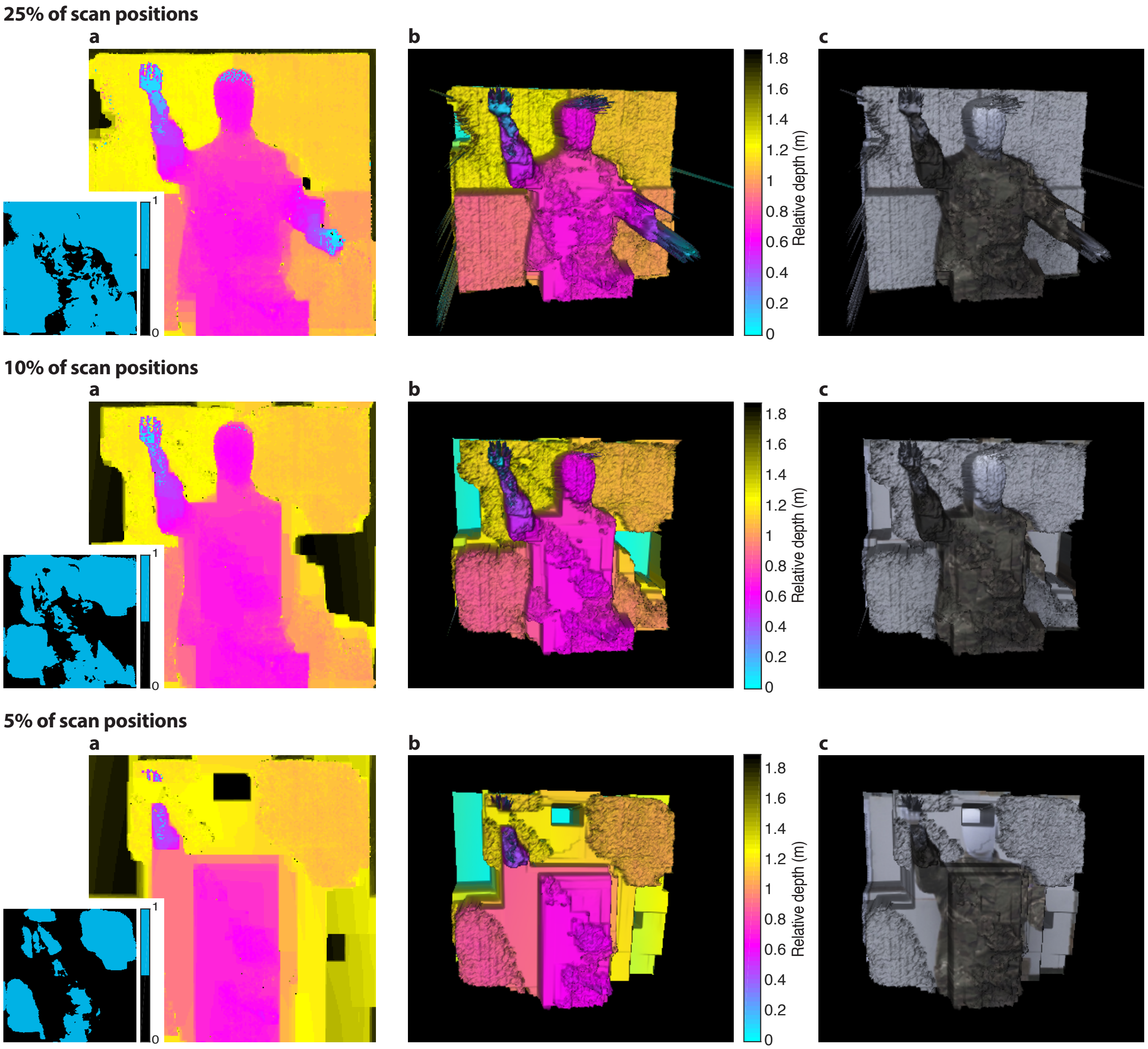

FIG. 4. Reconstructions of the target scene obtained by fitting with a TV prior using 25%, 10%, and 5% of the scanned positions. Retrieved depth information in (a) 2D and (b) 3D. (c) The intensity information from a DSLR camera overlaid on top of the retrieved depth information in 3D.

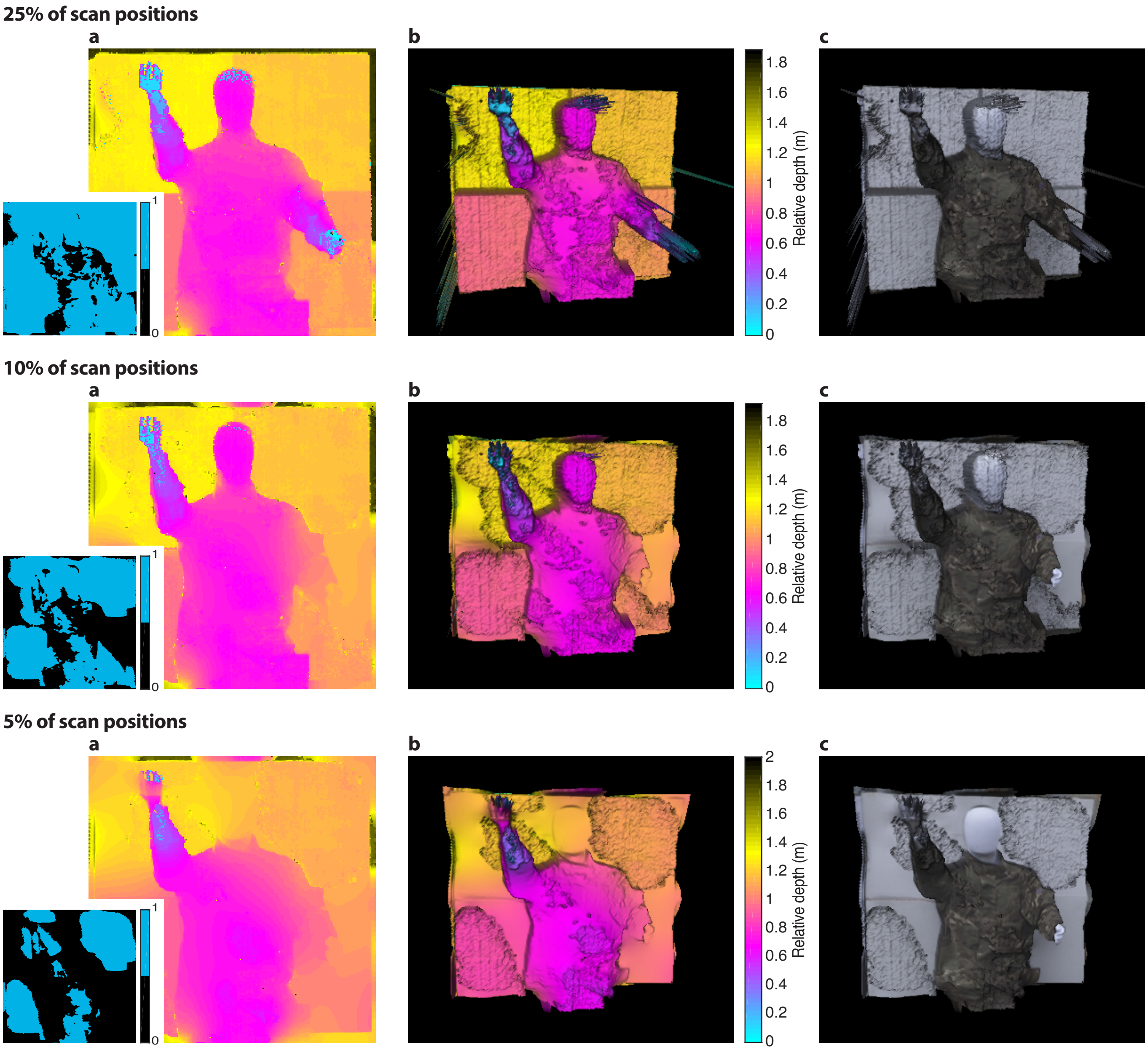

FIG. 5. Reconstructions of the target scene obtained by non-local data fusion using 25%, 10%, and 5% of the scanned positions. Retrieved depth information in (a) 2D and (b) 3D. (c) The intensity information from a DSLR camera overlaid on top of the retrieved depth information in 3D.
